# Pericardial irradiation dose may be strongly associated with grade 4 lymphopenia and affect prognosis in patients with locally advanced esophageal cancer receiving definitive concurrent chemoradiotherapy

**DOI:** 10.1111/1759-7714.15057

**Published:** 2023-07-30

**Authors:** Xianyan Chen, Xiaoman Tian, Xuanwei Zhang, Qianyue Deng, Min Wang, Xiaojuan Zhou, Yong Xu, Lin Zhou, Bingwen Zou, Yongmei Liu, Rui Ao, You Lu, Youling Gong

**Affiliations:** ^1^ Division of Thoracic Tumor Multidisciplinary Treatment, Cancer Center and State Key Laboratory of Biotherapy, West China Hospital Sichuan University Chengdu China; ^2^ Department of Oncology Sichuan Provincial People's Hospital Chengdu China

**Keywords:** concurrent chemoradiotherapy, immune‐checkpoint inhibitor, locally advanced esophageal cancer, lymphopenia, pericardial irradiation

## Abstract

**Background:**

The immune system may influence prognosis, and lymphopenia is a frequent side effect of concurrent chemoradiotherapy (CCRT). Radical irradiation for locally advanced esophageal cancer (LA‐EC) exposes significant vascular and heart volumes. In this study, we hypothesized that lymphopenia is linked to cardiac and pericardial doses and affects patient prognosis.

**Methods and Materials:**

We identified 190 LA‐EC patients who received radical CCRT. Multivariate analysis (MVA) was performed to correlate clinical factors and dosimetric parameters with overall survival (OS). We collected lymphocyte‐related variables and ratios before and during CCRT. MVA was performed to correlate hematologic toxicity with OS. The relationship between dosimetric parameters and G4 lymphopenia was determined using logistic stepwise regression. Finally, a nomogram of G4 lymphopenia was developed and validated externally.

**Results:**

Median follow‐up time for all patients was 27.5 months. On MVA for OS, higher pericardial V_30_ (PV_30_) was linked to worse survival (HR: 1.013, 95% CI: 1.001–1.026, *p* = 0.039). The median OS stratified by PV_30_ > 55.3% and PV_30_ ≤ 55.3% was 24.0 months and 54.0 months, respectively (*p* = 0.004). G4 lymphopenia was shown to be linked with worse OS in the MVA of hematological toxicity with OS (HR: 2.042, 95% CI: 1.335–3.126, *p* = 0.001). Thirty of the 100 patients in the training set had G4 lymphopenia. Logistic stepwise regression was used to identify variables associated with G4 lymphopenia, and the final model consisted of stage‐IVA (*p* = 0.017), platelet‐to‐lymphocyte ratio during CCRT (*p* = 0.008), Heart V50 (*p* = 0.046), and PV_30_ (*p* = 0.048). Finally, a nomogram predicting G4 lymphocytopenia were constructed and externally validated. The ROC curve showed an AUC for internal validation of 0.775 and external validation of 0.843.

**Conclusion:**

Higher doses of pericardial radiation might affect LA‐EC patients' prognosis by inducing G4 lymphopenia during CCRT. Further prospective studies are warranted to confirm these findings, especially in the era of immune‐checkpoint inhibitor treatment.

## Graphical abstract

In this study, we investigated the relationship between pericardial and cardiac radiation dosimetric parameters and lymphopenia. Since lymphocytes are extremely sensitive to radiation and the pericardium anatomically contains the heart and part of the large blood vessels, higher pericardial radiation doses are prone to cause circulating immune cell reduction. Since grade 4 (G4) lymphopenia is strongly associated with prognosis, we developed a nomogram model to predict G4.

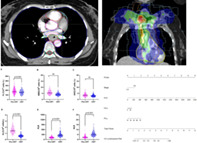

## INTRODUCTION

Radical concurrent chemoradiotherapy (CCRT) is the standard of care for patients with unresectable locally advanced esophageal cancer (LA‐EC). However, survival remains disappointing, with a 5‐year overall survival (OS) of only about 20%.^1^ The RTOG 0617 phase 3 clinical trial suggests that higher radiation doses may lead to poorer OS in patients with non‐small cell lung cancer (NSCLC).^2^ This may be because the delivery of high‐dose radiotherapy increases the risk of radiation‐induced injury to organs at risk (OARs), such as the heart, pericardium, and lungs.

Several studies have already been published which have explored the potential relationship between higher doses of irradiation to the heart and clinical outcomes for patients.^3^, ^4^, ^5^, ^6^ Very recently, the study by Cai et al. demonstrated that radiation exposure to the left anterior descending (LAD) and left circumflex (LCX) was strongly correlated with worse OS and was an excellent predictor of G3+ acute coronary syndrome/congestive heart failure.^7^

Myelosuppression is also a common side effect of antineoplastic therapy, and lymphocytopenia has been found to be significantly linked to patient treatment outcomes. Since lymphocytes are the most radiosensitive cells in the hematopoietic system, and the heart and lungs, as specific organs of the blood pool, receive high doses of radiation that may damage circulating lymphocytes.^8^, ^9^, ^10^ Several studies have shown that G4 lymphocytopenia during treatment is associated with poorer clinical outcomes in EC patients.^11^, ^12^ Prior studies have also shown that a low absolute lymphocyte count (ALC) during treatment is associated with poorer clinical outcomes, as has been demonstrated in lung, nasopharyngeal, liver, and rectal cancers.^13^, ^14^, ^15^, ^16^ So far, rarely available data report the impacts of irradiation dose to OARs on lymphocytes consequently influencing the prognosis of LA‐EC patients.

The primary objective of the present study was to investigate the relationship between radiation parameters and the risk of G4 lymphopenia in LA‐EC patients undergoing radical CCRT. Additionally, we assessed whether G4 lymphocyte reduction during CCRT was associated with patient prognosis and attempted to establish a nomogram for predicting G4 lymphocyte nadir.

## METHODS

### Patients

This retrospective study included 190 patients with LA‐EC (American Joint Committee on Cancer [AJCC] version 8, II–IVA stage) who received radical CCRT at West China Hospital and Sichuan Provincial People's Hospital between February 2011 and April 2021. The inclusion criteria were as follows: new pathologically verified ESCC with no distant metastases, no history of chest radiotherapy, and conventional segmentation at doses ≥50 Gy. Age > 75 years, a history of chest radiotherapy, surgical treatment, distant metastases, and a performance status (PS) score ≥2 were excluded from the study. Ethical approval was obtained from both institutions for this study, and the requirement for informed consent was waived due to its retrospective nature.

### Treatment and dosimetric evaluation

All patients underwent CCRT, with a radiation technique of intensity‐modulated radiation therapy (IMRT). The target volume contours were in accordance with the protocols in the study of Nishimura et al.^17^ The gross tumor volume (GTV) was calculated as the sum of the primary lesion (GTVp) and the metastatic lymph node volume (GTVn). The clinical target volume (CTV) was defined as the GTVp plus a 3 cm margin in the longitudinal direction of the esophagus and a 5 mm radial margin, and GTVn with a 5 mm expansion. The planning target volume (PTV) was defined as the CTV with a 5–10 mm expansion. Each patient received conventional fractionation therapy of 1.8–2.0 Gy with a minimum radiation dose of 50 Gy. The heart, pericardium, and lungs were included in the analysis. The heart and lungs were outlined by atlas‐based automated contouring software with necessary modifications based on the cardiac atlas published by Feng et al.^18^ Based on the RTOG 1106 organ at risk atlas, the pericardium was manually outlined by two investigators, starting 1–2 layers (5–6 mm) above the aortic arch and ending at the septum at the apex of the pericardium, which encloses the heart chambers, some large blood vessels, and adipose tissue. Next, the accuracy and consistency of each patient's structure were reviewed by an experienced radiophysicist. Dose‐volume histograms (DVH) were extracted from the Pinnacle or Raystation treatment planning system for the heart, pericardium, and lungs including the average dose, maximum dose, and the relative volume receiving the given dose (from ≥5 Gy to ≥60 Gy in 5 Gy increments). For normal tissues, the dose‐volume constraints were as follows: to the spinal cord, ≤45 Gy; to the heart, V_30_ (volume receiving 30 Gy) ≤ 30%, MHD < 30 Gy; and to the whole lung, V_20_ ≤ 20%, V_30_ ≤ 15% and MLD < 20 Gy as recommended by NCCN panels.^19^ Synchronous chemotherapy regimens usually consist of paclitaxel, fluoropyrimidine, and platinum‐based compounds.

### Hematological toxicities

We collected blood cell variables such as ALC, absolute platelet count (PLT), absolute white blood cell count (WBC), absolute neutrophil count (ANC), neutrophil‐to‐lymphocyte ratio (NLR = ANC/ALC), and platelet‐to‐lymphocyte ratio (PLR = PLT/ALC) 1 month before CCRT and weekly throughout CCRT. According to CTCAE5.0, G4 lymphopenia was defined as ALC nadir <0.2 10^3^/mL, which was used to dichotomize the lowest lymphocytes during CCRT.

### Statistical analysis

We made the following assumptions; first, higher pericardial, cardiac, and pulmonary doses were linked to poorer OS. The primary outcome in the first analysis was OS, defined as CCRT completion to all‐cause death or cutoff follow‐up time. Second, we hypothesized that severe lymphopenia was associated with higher cardiac and macrovascular radiation doses thus affecting prognosis. In the second analysis, the primary endpoint was G4 lymphopenia. Kaplan–Meier curves and log‐rank tests were used to compare the results of the G4 nadir. Stepwise backward logistic regression was used to find the association of clinical, hematological, and dosimetric variables with G4 lymphopenia. The variable with the lowest Akaike information criterion (AIC) value was selected for the actual modeling due to the strong covariance between cardiac and pericardial DVH parameters. The results of multivariate analyses were used to construct a nomogram. *p*‐values with two tails less than 0.05 were regarded as statistically significant. All analyses were performed using R software (version 4.2.2) and SPSS (version 26.0).

### Nomogram construction and external validation

A total of 100 from West China Hospital and 90 patients from Sichuan Provincial People's Hospital were included in the training and validation cohorts, respectively. Results of multivariate logistic regression analysis were used to construct a nomogram. Internal validation of the nomogram was performed using the area under the subject operating characteristic (ROC) curve (AUC), calibration curve (1000 bootstrap resampling), and decision curve analysis (DCA). The final prediction model was applied to the external cohort to generate the (externally validated) C‐index.

## RESULTS

### Patient characteristics

The baseline patient characteristics are summarized in Table 1. The median age at diagnosis was 64 years. The tumors were most commonly T3 (50%) with N1 (45%) lymph node status. Most tumors were located in the upper esophagus (49%); 60% were stage II–III, and 40% were stage IVA. The median prescribed dose was 58 Gy (range 54–60 Gy). The median follow‐up time for all patients was 27.5 months (range 12.0–118.0 months). The estimated median overall survival for all patients was 47.0 months (95% CI: 32.3–61.6).

**TABLE 1:** Baseline patient, tumor, and treatment characteristics (*n* = 190).

Characteristics	No. of patients (%)
Age, years, median	64 (34–74)
Sex	
Male	137 (72%)
Female	53 (28%)
ECOG PS	
0	155 (81%)
1	35 (19%)
Smoking	
Yes	118 (62%)
No	72 (38%)
Tumor location	
Upper thoracic	93 (49%)
Midthoracic	69 (36%)
Lower thoracic	28 (15%)
T status^a^	
T1	2 (1%)
T2	28 (15%)
T3	95 (50%)
T4	65 (34%)
N status^a^	
N0	36 (19%)
N1	86 (45%)
N2	50 (26%)
N3	18 (9%)
Stage^a^	
II–III	114 (60%)
IVA	76 (40%)
Radiation dose	
Dose (Gy)/fraction, median	2.0 (1.8–2.0)
Prescribed RT dose, median (IQR), Gy	58.0 (54.0–60.0)
Mean pericardium dose, median (IQR), Gy	28.1 (20.8–33.9)
Mean heart dose, median (IQR), Gy	20.3 (6.2–28.7)
Mean lung dose, median (IQR), Gy	12.5 (10.9–14.0)

Abbreviations: ECOG PS, Eastern Cooperative Oncology Group performance status; Gy, gray; IQR, interquartile range; RT, radiotherapy.

^a^ American Joint Committee on Cancer staging manual, eighth edition.

### Univariate and multivariate models of OS

**FIGURE 1:**
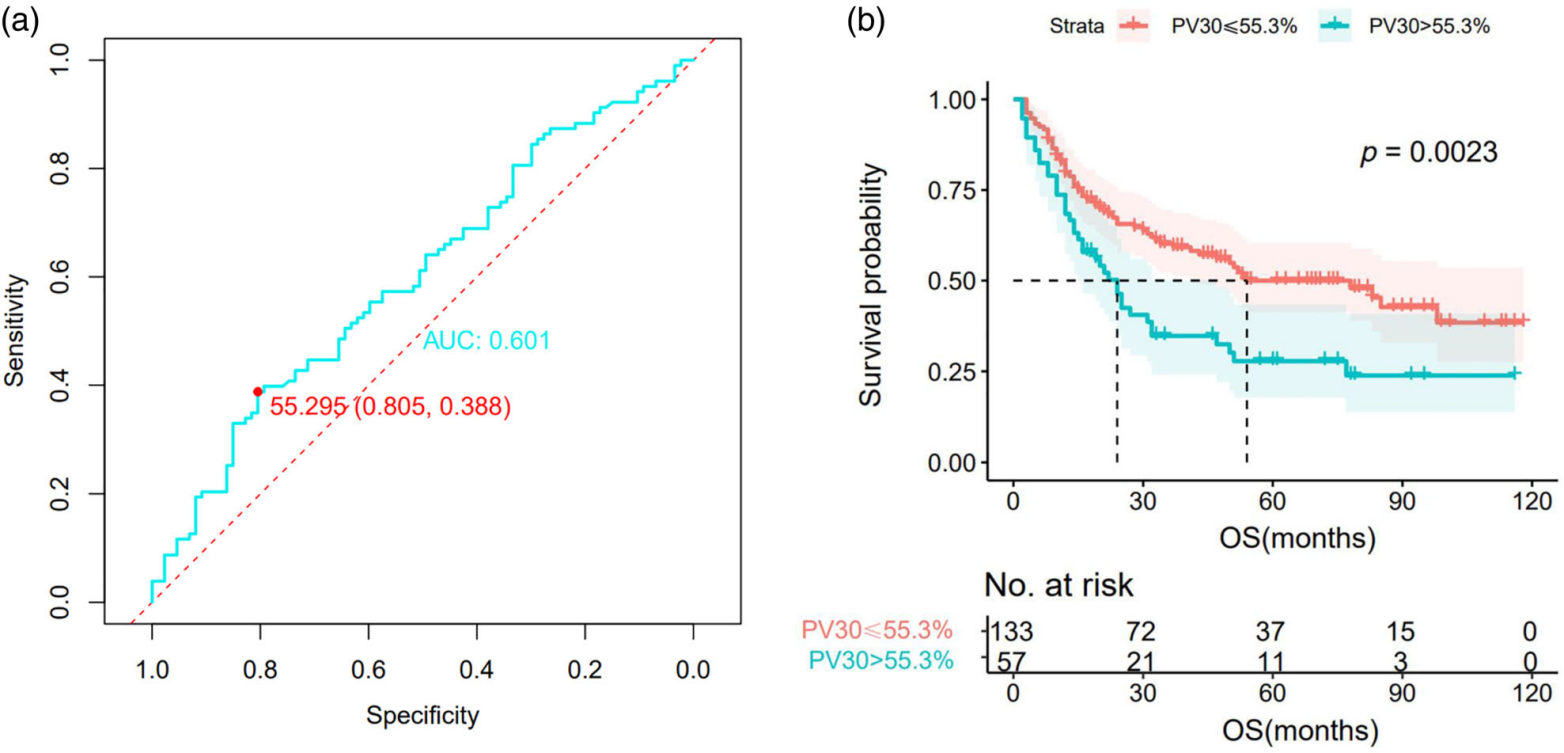
(a) The receiver operating characteristic (ROC) curve of PV_30_. (b) Survival curves for pericardium V_30_ > 55.3% versus V_30_ ≤ 55.3%.

The clinical and dosimetric factors associated with OS are summarized in Table 2. COX univariate analysis showed that stage‐IVA, pericardial V_25_ (PV_25_), and pericardial V_30_ (PV_30_) were associated with poorer OS (*p* < 0.05), while gender, age, smoking status, ECOG, prescribed dose, and tumor location were not significantly different. Notably, cardiac and pulmonary dosimetric parameters were not significantly associated with OS (Table 2). On MVA for OS, PV_30_ remained statistically significant (HR: 1.013, 95% CI: 1.001–1.026, *p* = 0.039). We plotted the ROC curve of PV_30_ versus OS and calculated the optimal cutoff for PV_30_ at 55.3% (Figure 1a). Dichotomizing PV_30_ with this threshold and plotting survival curves (Figure 1b). The median OS stratified by PV_30_ > 55.3% and PV_30_ ≤ 55.3% was 24.0 and 54.0 months, respectively (*p* = 0.004).

**TABLE 2:** Univariate and multivariate Cox regression of clinical and dosimetric factors with OS.

Variable		Univariate analysis		Multivariate analysis	
		HR (95% CI)	*p*‐value	HR (95% CI)	*p*‐value
Age, years	≥60 vs. <60	1.004 (0.98–1.03)	0.743	‐	‐
Sex	Female vs. male	0.881 (0.57–1.36)	0.567	‐	‐
Smoking	Yes vs. No	1.308 (0.87–1.96)	0.193	‐	‐
ECOG	1 vs. 0	1.30 (0.803–2.11)	0.286	‐	‐
Tumor location	Lower vs. upper /middle	1.173 (0.66–2.07)	0.580	‐	‐
T status	T3–4 vs. T1‐2	1.139 (0.659–1.97)	0.150	‐	‐
N status	N2–3 vs. N0‐1	1.047 (0.696–1.58)	0.125	‐	‐
Stage	IVA vs. III/II	1.203 (1.027–1.78)	0.047	‐	‐
Prescribed dose, Gy	Continuous	0.983 (0.934–1.03)	0.505	‐	‐
MHD, Gy	Continuous	1.011 (0.994–1.03)	0.205	‐	‐
HV_5_(%)	Continuous	1.003 (0.997–1.008)	0.360	‐	‐
HV_10_(%)	Continuous	1.003 (0.998–1.009)	0.269	‐	‐
HV_20_(%)	Continuous	1.004 (0.998–1.011)	0.195	‐	‐
HV_30_(%)	Continuous	1.007 (0.997–1.016)	0.161	‐	‐
MPD, Gy	Continuous	1.024 (0.999–1.05)	0.056	‐	‐
PV_5_(%)	Continuous	1.005 (0.997–1.013)	0.232	‐	‐
PV_10_(%)	Continuous	1.006 (0.998–1.014)	0.167	‐	‐
PV_20_(%)	Continuous	1.007 (0.998–1.017)	0.116	‐	‐
PV_25_(%)	Continous	1.011 (1.001–1.023)	0.040	‐	‐
PV_30_(%)	Continous	1.013 (1.001–1.026)	0.039	1.013 (1.001–1.026)	0.039
MLD, Gy	Continuous	0.993 (0.968–1.018)	0.558	‐	‐
LV_5_(%)	Continuous	1.001 (0.986–1.016)	0.903	‐	‐
LV_10_(%)	Continuous	1.000 (0.979–1.020)	0.964	‐	‐
LV_20_(%)	Continuous	1.001 (0.962–1.041)	0.975	‐	‐
LV_30_(%)	Continuous	1.012 (0.954–1.073)	0.688	‐	‐

Abbreviations: CI, confidence interval; ECOG, Eastern Cooperative Oncology Group; Gy, gray; HR, hazard ratio; MHD, mean heart dose; MLD, mean lung dose; MPD, mean pericardial dose; V_x_, percent volume receiving X Gy.

### Hematological toxicity with worse OS

**FIGURE 2:**
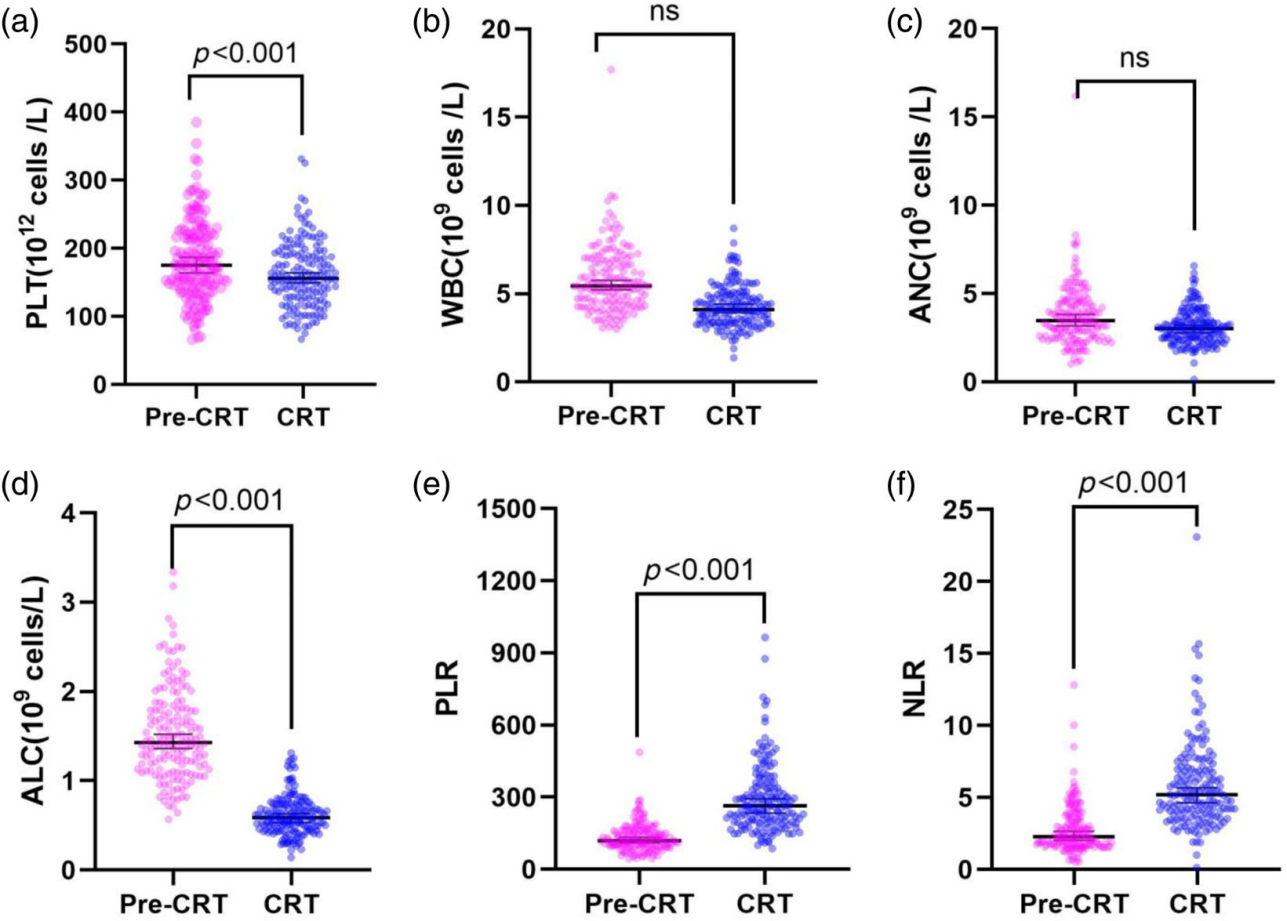
Grape plot of blood cell count changes. (a) Absolute platelet count (PLT) prechemoradiotherapy (CRT) and during CRT. (b) White blood cell count (WBC) pre‐CRT and during CRT. (c) Absolute neutrophil count (ANC) pre‐CRT and during CRT. (d) Absolute lymphocyte count (ALC) pre‐CRT and during CRT. (e) Platelet‐to‐lymphocyte ratio (PLR) pre‐CRT and during CRT. (f) Neutrophil‐to‐lymphocyte ratio (NLR) pre‐CRT and during CRT.

The median baseline ALC (ALC‐b), baseline PLT (PLT‐b), baseline PLR (PLR‐b), and baseline NLR (NLR‐b) before chemoradiotherapy (CRT) were 1.43 × 10^9^ cells/L, 175.2 × 10^12^ cells /L, 119.67, and 2.275, respectively. The median ALC during CRT, PLT, PLR, and NLR during CRT were 0.59 × 10^9^ cells/L, 156 × 10^12^ cells /L, 264.17, and 5.19, respectively. The median lymphocyte nadir during CRT was 0.27 × 10^9^ cells/L (range: 0.04–0.78). At the nadir, all patients experienced grade 2 or higher lymphopenia, with 111/190 (58%) cases having grade 3 lymphopenia and 58/190 (31%) grade 4 lymphopenia. We plotted the comparative blood cell changes before and after CRT (Figure 2). As expected, PLT and ALC were significantly reduced during CRT compared to pre‐CRT (*p* < 0.001). NLR and PLR were significantly higher (*p* < 0.001). However, WBC and ANC were not significantly different. Table 3 summarizes the univariate and multivariate models of blood cell and OS‐related factors. G4 lymphocytopenia was associated with poorer OS (HR: 2.042, 95% CI: 1.335–3.126, *p* = 0.001). The median OS was 19.0 and 54.0 months for G4 and non‐G4 groups, respectively (*p* = 0.001).

**TABLE 3:** Univariate and multivariate Cox regression of hematological toxicity with OS.

Variable	Univariate analysis		Multivariate analysis	
	HR (95% CI)	*p*‐value	HR (95% CI)	*p*‐value
Pre‐CRT				
WBC‐b	0.862 (0.756–0.982)	0.026	‐	‐
PLT‐b	1.001 (0.998–1.005)	0.443	‐	‐
ALC‐b	0.854 (0.579–1.261)	0.427	‐	‐
ANC‐b	0.847 (0.728–0.986)	0.032	‐	‐
PLR‐b	1.001 (0.997–1.004)	0.747	‐	‐
NLR‐b	0.881 (0.765–1.014)	0.077	‐	‐
During‐CRT				
WBC	0.999 (0.840–1.188)	0.993	‐	‐
PLT	1.000 (0.996–1.005)	0.899	‐	‐
ALC	1.430 (0.574–3.562)	0.442	‐	‐
ANC	1.014 (0.828–1.242)	0.894	‐	‐
PLR	1.000 (0.998–1.001)	0.437	‐	‐
NLR	0.982 (0.914–1.054)	0.612	‐	‐
G4 lymphopenia (Yes vs. No)	2.042 (1.335–3.126)	0.001	2.042 (1.335–3.126)	0.001

Abbreviations: ALC‐b, baseline absolute lymphocyte count; ANC‐b, baseline absolute neutrophil count; CI, confidence interval; CRT, chemoradiotherapy; HR, hazard ratio; G4, grade 4; NLR, neutrophil to lymphocyte ratio; OS, overall survival; PLR, platelet to lymphocyte ratio; PLT‐b, baseline absolute platelet count; WBC‐b, baseline white blood cell count.

### Univariate and multivariate models of G4 lymphopenia

Next, we used a training group of 100 patients from West China Hospital, 30 of whom had G4 lymphopenia. Logistic univariate and multivariate analyses were performed to identify the factors associated with G4 lymphopenia (Table 4). In the univariate analysis, gender (female), stage (IVA), PLR‐b, and PLR, NLR, MHD, HV_5_‐HV_55_, MPD, and PV_5_‐PV_35_ were associated with G4 lymphopenia. Next, stepwise backward regression was performed to select the variable with the lowest Akaike information criterion (AIC) value to establish the model. The final prediction models were constructed by stage‐IVA (*p* = 0.017), PLR (*p* = 0.008), HV_50_ (*p* = 0.046), and PV_30_ (*p* = 0.048).

**TABLE 4:** Univariate and multivariate logistic regression analysis of G4 nadir.

Variable	Univariate analysis		Multivariate analysis	
	OR (95% CI)	*p*‐value	OR (95% CI)	*p*‐value
Age, years	0.987 (0.930–1.046)	0.654	‐	‐
Sex Female vs. male	0.862 (0.756–0.982)	0.026	‐	‐
Smoking Yes vs. No	1.103 (0.422–2.882)	0.841	‐	‐
ECOG 1 vs. 0	0.911 (0.220–3.771)	0.897	‐	‐
Tumor location Lower vs. upper /middle	1.767 (0.451–6.923)	0.414	‐	‐
Stage IVA vs. III/II	3.619 (1.329–9.859)	0.012	4.493 (1.373–16.963)	0.017
ALC‐b, 10^9^ cells/L	0.482 (0.186–1.252)	0.134	‐	‐
PLR‐b	1.010 (1.001–1.020)	0.027	‐	‐
NLR‐b	1.258 (0.921–1.717)	0.149	‐	‐
ALC, 10^9^ cells/L	0.048 (0.003–0.681)	0.025	‐	‐
PLR	1.006 (1.002–1.011)	0.006	1.007 (1.002–1.014)	0.008
NLR	1.363 (1.112–1.669)	0.003		
Prescribed dose, Gy	1.001 (0.997–1.004)	0.747	‐	‐
MHD, Gy	1.045 (1.002–1.090)	0.039	‐	‐
HV_5_(%)	1.013 (1.000–1.027)	0.057		
HV_10_(%)	1.015 (1.001–1.029)	0.033	‐	‐
HV_15_(%)	1.016 (1.001–1.030)	0.036	‐	‐
HV_20_(%)	1.017 (1.001–1.033)	0.041	‐	‐
HV_25_(%)	1.020 (1.001–1.039)	0.042	‐	‐
HV_30_(%)	1.025 (1.001–1.049)	0.040	‐	‐
HV_35_(%)	1.033 (1.002–1.065)	0.039	‐	‐
HV_40_(%)	1.044 (1.001–1.089)	0.042	‐	‐
HV_45_(%)	1.063 (1.003–1.127)	0.040	‐	‐
HV_50_(%)	1.111 (1.018–1.212)	0.018	1.524 (1.032–2.709)	0.046
HV_55_(%)	1.201 (1.049–1.376)	0.015	‐	‐
MPD, Gy	1.074 (1.008–1.145)	0.029	‐	‐
PV_5_(%)	1.023 (1.003–1.045)	0.027	‐	‐
PV_10_(%)	1.027 (1.005–1.049)	0.014	‐	‐
PV_15_(%)	1.028 (1.006–1.052)	0.014	‐	‐
PV_20_(%)	1.031 (1.006–1.057)	0.015	‐	‐
PV_25_(%)	1.036 (1.005–1.068)	0.021	‐	‐
PV_30_(%)	1.048 (1.001–1.087)	0.011	1.172 (1.014–1.399)	0.048
PV_35_(%)	1.056 (1.010–1.104)	0.017	‐	‐
MLD, Gy	0.975 (0.898–1.058)	0.542	‐	‐
LV_5_(%)	1.039 (0.998–1.082)	0.065	‐	‐
LV_10_(%)	1.053 (0.996–1.112)	0.070	‐	‐
LV_20_(%)	1.079 (0.953–1.221)	0.229	‐	‐
LV_30_(%)	0.963 (0.818–1.134)	0.652	‐	‐

Abbreviations: ALC‐b, baseline absolute lymphocyte count; CI, confidence interval; G4, grade 4; Gy, gray; MHD, mean heart dose; MLD, mean lung dose; MPD, mean pericardial dose; NLR‐b, baseline neutrophil‐to‐lymphocyte ratio; OR, odds ratio; PLR‐b, baseline platelet‐to‐lymphocyte ratio; V_x_, percent volume receiving X Gy.

### Development and validation of a nomogram

**FIGURE 3:**
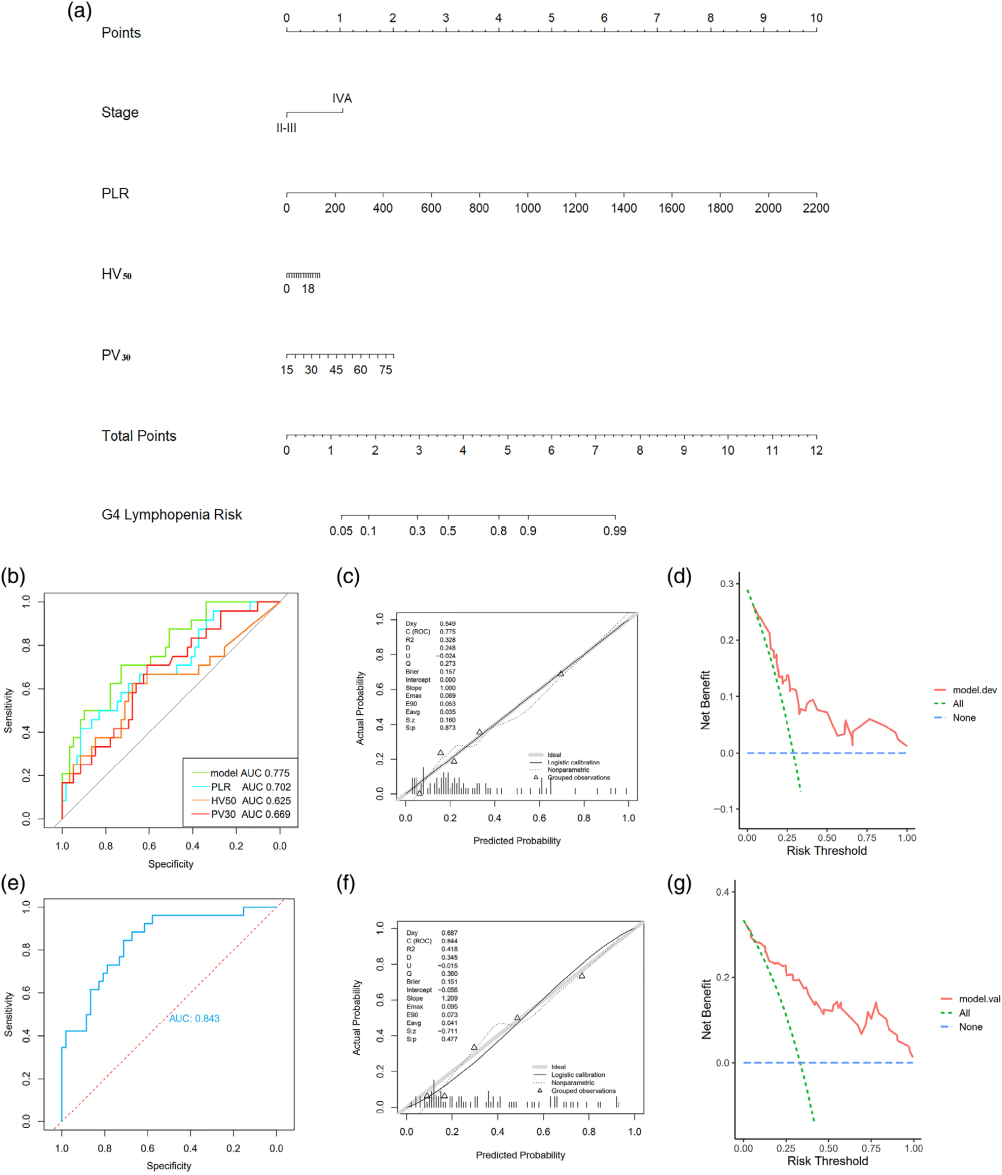
A nomogram for predicting G4 and internal and external validation. (a) Nomogram predicting the occurrence of G4 nadir. For each patient, four lines are drawn upward to determine the points received from the four variables in the nomogram. The sum of these points is located on the “total points” axis, and a line is drawn downward to determine the likelihood of this patient having a G4 nadir. (b) Receiver operating characteristic (ROC) curves of platelet‐to‐lymphocyte ratio (PLR), HV_50_, PV_30_, and the predictive mode in the training dataset. (c) Calibration curves of the nomogram predicting the occurrence of G4 nadir in the training dataset. A perfect prediction would correspond to a slope of 1 (diagonal 45‐degree broken line). (d) Decision curves of the nomogram predicting the occurrence of G4 nadir in the training dataset. (e) ROC curve of external validation. (f) Calibration curves of external validation. (g) Decision curves of external validation.

Based on the multivariate logistic regression coefficients, the prediction model was presented as a nomogram (Figure 3a). In the scoring system, stage‐IVA corresponded to 1 point. A PLR value of 1000 corresponded to 4.5 points (higher PLR values result in higher scores). An HV_50_ value of 18% scored less than 1 point, while a PV_30_ value of 75% corresponded to 2 points. The total score was the sum of the above scores and corresponded to the percentage of risk below. The higher the total score, the higher the risk of developing G4 lymphopenia. To validate the model accuracy, we plotted the ROC curve of the PLR during CRT, PV_30_, HV_50_, and the prediction model (Figure 3b). The AUC of the final prediction model ROC curve was 0.775. For external validation, 28 out of 90 patients had G4 lymphocytopenia. The AUC of the final external cohort ROC curve was 0.843 (Figure 3e). Both calibration and decision curves for internal and external validation showed favorable agreement between predicted and actual observed values (Figure 3c,d,f,g).

## DISCUSSION

According to the NCCN guidelines for esophageal cancer, OARs are recommended with dose‐volume constraints of HV_30_ ≤ 30% and MHD < 30 Gy; LV_20_ ≤ 20%, LV_30_ ≤ 15%, and MLD < 20 Gy and are applied worldwide in radiotherapy treatment planning.^19^ However, even when the above dose limits are met, there can be a significant difference in patient survival. Studies have shown that higher doses of certain OARs, such as the heart and lungs, are associated with poorer overall survival.^2^, ^4^, ^20^ For the first time, our study demonstrated that the higher pericardial irradiation dose (PV_30_) was strongly linked to G4 lymphopenia and might consequently affect prognosis in LA‐EC patients undergoing CCRT. The study also developed a nomogram for predicting the occurrence of G4 lymphopenia, which showed excellent predictive value in both internal and external validation.

Extensive studies have shown that higher cardiac radiation doses were significantly associated with cardiotoxicity, which can affect clinical outcomes.^21^, ^22^ This is also why cancer treatment‐related cardiovascular toxicity (CTR‐CVT), including cardiomyopathy and heart failure (HF), myocarditis, vascular toxicity, hypertension, arrhythmia, and others, has received increasing attention in recent years.^23^ However, radiological cardiac complications were usually defined as late toxicity, occurring beyond 10 years of treatment in long‐term survivors of breast cancer and Hodgkin's lymphoma.^24^, ^25^, ^26^ Given the dismal overall survival rate of esophageal cancer patients, with a 5‐year OS of ~20%, making long‐term follow‐up of radiation‐induced cardiotoxicity challenging.

Lymphocytes are known to be extremely sensitive to radiation, with D50 (the dose required to inactivate 50% of the cells) as low as 2 Gy.^27^ Prior studies have shown that the radiation doses to specific organs of the blood pool (such as the lung and heart) may have a significant effect on circulating lymphocyte counts. For example, Xu et al. indicated that G4 lymphopenia during CCRT was associated with higher lung V_10_ and heart V_10_ and poorer survival.^11^ Another recent study conducted by the Shanghai Proton and Heavy Ion Center showed that severe lymphopenia was associated with aortic V_5_ and affected prognosis.^28^ Recently, a study that included 110 ESCC receiving IMRT showed that radiation‐induced lymphopenia (RIL) was associated with cardiac and pulmonary dose parameters, and the results also demonstrated that RIL could predict survival and radiation pneumonia.^29^ A meta‐analysis also revealed that RIL was associated with dosimetric factors such as PTV, cardiac dose, and effective dose to immune cells (EDIC), and it was correlated with lower pCR rates, as well as poorer OS and progression‐free survival (PFS).^30^ However, none of them analyzed the pericardial dose. Given the pericardium includes all cardiac chambers and some large vessels according to the RTOG 1106 risk organ atlas, it is reasonable to speculate that the higher pericardial radiation dose may have a negative impact on the immune system, thereby affecting patient outcomes. Similarly, a small prospective study of NSCLC demonstrated that higher pericardial doses (PV_30_ and PV_55_) were significantly associated with OS, but the mechanism was not further explored.^20^ Our analysis revealed that severe lymphopenia was highly associated with pericardial dosimetric parameters and that G4 nadir was linked to poorer outcomes, in line with the results of Kroese et al.^12^ A study conducted at the MD Anderson Cancer Center also reported that G4 lymphopenia was associated with poorer OS, but lymphocyte recovery did not affect prognosis, likely due to the inability of newly generated naive T lymphocytes to produce antitumor effects after lymphocyte recovery.^31^ Therefore, the predictive value of lymphocyte recovery was not explored further in our study. In addition, Xu et al. developed a model of EDIC for predicting the severity of radiation‐induced lymphopenia, which was calculated based on MLD, MHD, mean liver dose, and the integrated dose of the scanned body region.^32^ In our opinion, its relatively complex formulae were not convenient for practical clinical work. Meanwhile, the dose‐volume parameters of the pericardium are relatively easily focused and facilitate their application in practice. In our study, higher pericardial radiation doses showed excellent predictive value in a model of G4 lymphopenia and correlated with patient prognosis.

T lymphocytes play a central role in the immune system, as CD8+ and CD4+ T‐cells can directly damage tumor cells or generate cytokines that activate effector cells, thereby improving the prognosis of EC patients.^33^ G4 lymphocytopenia during CCRT is strongly associated with OS.^12^, ^31^ For locally advanced tumors, such as NSCLC, the PACIFIC study showed that radiotherapy (chemoradiotherapy) followed by immune maintenance was highly successful in stage III NSCLC, providing strong evidence‐based medical support for the combination of immunotherapy and radiotherapy for sensitization.^34^ The dynamics of circulating lymphocytes during CCRT have been shown to support earlier administration of anti‐PD‐1/PD‐L1 therapy and correlate with treatment outcomes. Currently, the clinical trials of CCRT followed by immune maintenance therapy are ongoing in inoperable esophageal cancer (https://clinicaltrials.gov identifier: NCT 04210115 and NCT 03957590), which may become the standard treatment modality for LA‐EC in the future. We eagerly await their updates on clinical advantages, treatment‐related toxicity, and other translational work.

Our study had some limitations. First, due to its retrospective nature, this study was subject to selective bias. Second, the sample size in this study was relatively small as we only included patients who received radical CCRT and IMRT under strict inclusion criteria. In several similar studies, clinical factors such as chemotherapy (sequential/concurrent) and RT techniques (protons/IMRT) might have impacts on treatment outcomes for these LA‐EC patients. Additionally, even though we recorded blood counts weekly, as recommended by the NCCN guidelines,^19^ it is possible that patients did not record at the nadir, so we could not obtain the actual nadir of ALC during CCRT precisely. Lastly, there was a strong correlation between the dosimetric parameters of the heart and pericardium, so we chose the variable with the lowest AIC (a measure of statistical model fit) value for modeling.^35^ Our findings suggest that limiting the radiation dose to the pericardium might minimize the incidence of G4 nadir compared to the heart. In the future, larger studies are necessary to investigate large vessel dosing and assess dosimetric factors associated with immunosuppression.

In conclusion, higher pericardial radiation doses are strongly associated with G4 nadir and affect the prognosis of LA‐EC patients undergoing radical CCRT. In the era of immune checkpoint inhibitor therapy, optimization of RT regimens to conserve the immune system may be an important direction to improve prognosis.

## AUTHOR CONTRIBUTIONS

Youling Gong conceived and designed the study. Xianyan Chen, Xiaoman Tian, Xuanwei Zhang, Qianyue Deng and Min Wang collected the data. Xianyan Chen and Youling Gong analyzed and interpreted the data and drafted the article. Yong Xu, Lin Zhou, Xiaojuan Zhou, Yongmei Liu, Bingwen Zou, Rui Ao, You Lu critically revised the manuscript. All the authors approved the final submitted version.

## FUNDING INFORMATION

The authors declare that no funds, grants, or other support were received during the preparation of this manuscript.

## CONFLICT OF INTEREST STATEMENT

The authors declare no conflicts of interest.
